# Circular RNA hsa_circ_0064559 affects tumor cell growth and progression of colorectal cancer

**DOI:** 10.1186/s12957-023-03050-5

**Published:** 2023-06-07

**Authors:** Ya’nan Zhen, Guodong Sun, Cunbao Chen, Jianqi Li, Ruixue Xiao, Zhongfa Xu

**Affiliations:** 1 Department of Gastrointestinal Surgery, Shandong Provincial Third Hospital, Jinan, Shandong China; 2 Department of Gastrointestinal Surgery, The Third Affiliated Hospital of Shandong First Medical University (Affiliated Hospital of Shandong Academy of Medical Sciences), Jinan, Shandong China; 3 Gastroenterology Research Institute and Clinical Center, Shandong Academy of Medical Sciences), Shandong First Medical University, Jinan, Shandong China; 4 Traditional Chinese Medicine Department, The Third Affiliated Hospital of Shandong First Medical University (Affiliated Hospital of Shandong Academy of Medical Sciences), Jinan, Shandong China; 5 Department of Pathology, Shandong Provincial Third Hospital, Jinan, Shandong China

**Keywords:** Circular RNA, hsa_circ_0064559, Colorectal cancer, Proliferation, Apoptosis

## Abstract

**Background:**

Colorectal cancer (CRC) is the second leading cause of cancer-related deaths globally. It is essential to identify new CRC-associated therapeutic targets and diagnostic biomarkers. Previous studies have demonstrated that a series of circular RNAs (circRNAs) play a crucial role in CRC pathogenesis. This study assessed the potential of hsa_circ_0064559 in tumor cell growth and progression of CRC.

**Methods:**

Six pairs of matched CRC and normal colorectal tissue samples were sequenced using the Affymetrix Clariom D array. Using RNA interference, the expression of thirteen circRNAs was knocked down in CRC cells. The proliferation of CRC cell lines (RKO and SW620 cells) was detected using 3-(4,5-dimethyl-2-thiazolyl)-2,5-diphenyl-2-H-tetrazolium bromide (MTT) assay. Apoptosis and cell cycle were determined by flow-cytometric analysis. An in vivo study uses nude mice to establish a CRC mouse model. The differentially expressed genes were analyzed using Affymetrix primeview human GeneChip array and verified by polymerase chain reaction.

**Results:**

Affymetrix Clariom D array analysis revealed that thirteen circRNAs were upregulated in CRC. The proliferation of CRC cell lines was decreased, while the proportion of apoptotic and G1 phase cells was higher after hsa_circ_0064559 knockdown. In vivo xenograft nude mice model revealed that the volume and weight of the tumor were reduced by hsa_circ_0064559 knockdown. In Affymetrix primeview human GeneChip array, we found six upregulated genes (STAT1, ATF2, TNFRSF10B, TGFBR2, BAX, and SQSTM1) and two downregulated genes (SLC4A7 and CD274) related to apoptosis and proliferation of colorectal cancer cells after hsa_circ_0064559 knockdown.

**Conclusions:**

The hsa_circ_0064559 knockdown could inhibit the proliferation, promote apoptosis in CRC cell lines in vitro, and inhibit the development of CRC tumors in vivo. The mechanism may be related to activating a wide range of signaling pathways. The hsa_circ_0064559 may be a potential biomarker for early diagnosis or prognosis of CRC and a novel drug target for CRC therapy.

## Introduction

With 1.8 million new cases and more than 860,000 fatalities annually, colorectal cancer (CRC) is the second most prevalent cancer-related mortality and the fourth most common disease overall [1]. CRC is China’s most commonly diagnosed cancer and a significant public health concern [1]. High-fat diets and an aging population increased CRC incidence and mortality [2, 3]. A series of risk factors for CRC, such as obesity, alcohol consumption, smoking, and physical inactivity, have been investigated in various populations [4–6]. However, an upward trend in incidence has been reported in most developing countries, and the etiology of CRC still needs to be better understood [7–9]. Despite continuous advancements in diagnosis and treatment approaches, CRC remains the primary cause of death. Currently, biomarkers are indispensable in the diagnosis and treatment of CRC patients. Therefore, discovering new CRC-associated therapeutic targets and diagnostic biomarkers will facilitate the early detection and effective treatment of CRC.

As a class of evolutionarily conserved noncoding RNA, circular RNAs (circRNAs) form nonlinear closed-loop structures via bonds between their two ends, making them stable [10]. Structure stability prevents circRNAs from being easily degraded by RNA exonuclease and acting as a long-lasting transcriptional regulator. With the development of high-throughput microarray and sequencing technologies [11], the expression pattern of circRNAs has been unveiled in various pathological conditions [12, 13]. Growing evidence has indicated the association between aberrant circRNAs profile and tumor progression, metastasis, and recurrence [14–16]. For instance, some circRNAs promote proliferation, invasion, and metastasis, while evading apoptosis in renal cell carcinoma, hepatocellular carcinoma (HCC), non-small cell lung cancer, and CRC [17]. Moreover, the expression of circRNAs is dynamic during the chemotherapy of cancer patients; therefore, detecting their expression in blood and urine can predict tumor progression [18]. Thus, discovering cancer-related circRNAs may contribute to early diagnosis and prognostic evaluation of various cancers. However, circRNAs involved in the development and progression of CRC have yet to be investigated.

This study identified that hsa_circ_0064559 was involved in cell growth, apoptosis, cell cycle, and tumor development of CRC. These findings revealed a crucial role of hsa_circ_0064559 as a potential regulatory factor in the tumorigenesis and progression of CRC.

## Materials and methods

### Tissue specimens

Six pairs of fresh tumors and their adjacent (at least 5 cm away) normal tissues were collected from CRC patients who underwent surgical resection between 2016 and 2018 in the Third Affiliated Hospital of Shandong First Medical University (Affiliated Hospital of Shandong Academy of Medical Sciences). No patient had radiotherapy or chemotherapy before the operation. According to the pathological stage, the patients were divided into groups A (T2N0M0, I) and B (T4aN2M0, IIIC). The detailed baseline characteristics of patients were retrieved from the electronic medical records. The clinicopathologic characteristics of patients are summarized in Table 1. This study was approved by the ethics committee of the Third Affiliated Hospital of Shandong First Medical University (Affiliated Hospital of Shandong Academy of Medical Sciences) (approval no. FY2019005). Written informed consent was obtained from each subject at recruitment.

**Table 1:** The clinicopathological characteristics of the patients

ID	Age	Gender	Histology	Differentiation	TNM Stage	Anatomic site	Group
1	64	Female	Adenocarcinoma	Moderate	T_2_N_0_M_0_, I	Rectum	A
2	63	Female	Adenocarcinoma	Moderate	T_2_N_0_M_0_, I	Rectum	A
3	55	Male	Adenocarcinoma	Moderate	T_2_N_0_M_0_, I	Rectum	A
4	58	Male	Adenocarcinoma	Moderate	T_4a_N_2_M_0_, IIIC	Rectum	B
5	55	Male	Adenocarcinoma	Moderate -low	T_4a_N_2_M_0_, IIIC	Rectum	B
6	54	Female	Adenocarcinoma	Moderate -low	T_4a_N_2_M_0_, IIIC	Rectum	B

*TNM* Tumor-node-metastasis

### CRC cell lines

The CRC cell lines, RKO and SW620, were used in our study. The RKO cells were purchased from the American Type Culture Collection (Manassas, USA), and SW620 cells were obtained from GeneChem Co. Ltd. (Shanghai, China). The cells were cultured in Dulbecco’s modified Eagle medium (DMEM) (Gibco, Waltham, MA, USA) with 10% fetal bovine serum (Gibco, Waltham, MA, USA), 100 U/mL penicillin G, and 100 μg/mL streptomycin (Sigma, Shanghai, China) at 37 °C in a humid incubator with 5% CO_2_.

### RNA interference

Short hairpin RNA targets for hsa_circ_0064559 (shCIRC) and empty vectors (shEV) were synthesized by GeneChem Co. Ltd. (Shanghai, China). The sequence of shCIRC was 5′-TTTATTATAGCCTGCCACAGC-3′, and shEV was 5′-TTCTCCGAACGTGTCACGT-3′. Lipofectamine 2000 (Invitrogen, Shanghai, China) was used for transfection in accordance with the manufacturer’s instructions. The transfection efficiency of cells was examined under a fluorescence microscope after 24 h. The cells were incubated for fifteen days to produce stably transfected cells (with transfection efficiency > 50%). Quantitative real-time polymerase chain reaction (qRT-PCR) verified the efficiency of knockdown.

### High-content screening (HCS) and cell growth curve analysis

We assessed the development of cultured cells using multiparametric HCS. The cells were seeded in 96-well plates in 100 µL of media 72 h after shRNA transduction at a density of 2000 cells per well. HCS was used to initially screen genes with obvious proliferation inhibitory phenotype by comparing the impact of gene knockdown on cell proliferation using Celigo (Nexcelom, Beijing, China). To ensure gene interference efficiency, we designed three RNA interference targets for each gene and mixed three grains with different targets for mixed virus packaging to ensure gene knockdown efficiency. According to the expressed fluorescent protein signal after the cells were infected, their proliferation can be observed according to the number of cells. The Cellomics ArrayScan VTI high-content image analysis platform was used to monitor cell growth daily for 5 days. HCS Studio Cell Analysis Software was used to analyze the data (Thermo Fisher Scientific, Waltham, MA, USA).

### qRT‑PCR

TRIzol reagent (Thermo Fisher Scientific, Waltham, MA, USA) was used to extract total RNA from cells (Invitrogen, Carlsbad, CA, USA). NanoDrop 2000 spectrophotometer was used to measure RNA concentration (Thermo Fisher Scientific, Rockford, IL, USA). First-strand cDNA synthesis kits (Invitrogen, Carlsbad, CA, USA) were used to reverse transcribe 20–100 ng RNA, as directed by the manufacturer. The mRNA expression levels were determined using qRT-PCR using an ABI 7500 System (Applied Biosystems, Foster City, CA, USA). Glyceraldehyde-3-phosphate dehydrogenase (GAPDH) was used as an internal control to determine the relative expression levels of genes. Three independent experiments were performed. The primer sequences (from Shanghai GeneChem Co., Ltd., Shanghai, China) used are shown in Table 2.

**Table 2:** The primer sequences used in this study

Gene	Forward primer sequence	Reverse primer sequence
GAPDH	TGACTTCAACAGCGACACCCA	CACCCTGTTGCTGTAGCCAAA
hsa_circ_0064559	TTTATTGGCAATGACGACCTG	GGAAGGATGGAGGGAGAAAGG
STAT1	GGCACCAGAACGAATGAGG	CCACAACGGGCAGAGAGG
ATF2	GTCATGGTAGCGGATTGGTTA	CTTTGGGTCTGTGGAGTTGTG
TNFRSF10B	TCACAGTTGCAGCCGTAGTC	TGTGAGCTTCTGTCCACACG
TGFBR2	GTGCCAACAACATCAACC	GACTGCCACTGTCTCAAACT
BAX	TGCTTCAGGGTTTCATCCA	GGCCTTGAGCACCAGTTT
SQSTM1	GAGTCGGATAACTGTTCAGGAGG	CGGATTCTGGCATCTGTAGGG
SLC4A7	ACATTCTGACCCTCACTTGCT	TTCCACCACTTCCATTACCTT
CD274	ACTGGCATTTGCTGAACG	TCCTCCATTTCCCAATAGAC

### Microarray and bioinformatics analysis

We identified differentially expressed genes in colorectal tumor tissues and matched adjacent normal tissues using Affymetrix Clariom D array (CNKINGBIO Co.Ltd., Beijing, China), and between shEV and shCIRC RKO cells using Affymetrix GeneChip primeview human gene expression array (GeneChem Co.Ltd., Shanghai, China). The hierarchical clustering and R software (version 4.1.2, https://www.r-project.org) performed quantile normalization and data processing. The sequencing data were deposited in NCBI’s Gene Expression Omnibus and are accessible through the GEO accession numbers GSE209892 and GSE226489.

### Cell proliferation assay

After lentiviral transduction, cell viability was determined using a 3-(4,5-dimethyl-2-thiazolyl)-2,5-diphenyl-2-H-tetrazolium bromide (MTT) assay. Briefly, logarithmic growth phase cells were seeded in 96-well plates at a 1.0 × 10^4^ cells/mL density in 200 μL media and incubated at 37 °C in a 5% CO_2_ atmosphere. The medium was replaced with serum-free DMEM containing 1 mg/mL MTT at 24, 48, 72, 96, and 120 h, respectively. The cells were cultured for an additional 4 h at 37 °C. After removing the supernatant from each well, 150 μL of dimethyl sulfoxide was added to all wells. After 10 min of gentle vortexing and agitation, optical density values at 490 nm were detected using a microplate reader (Bio-Rad Laboratories Inc., Hercules, CA, USA).

### Cell apoptosis assay

Annexin V-APC Apoptosis Detection Kit (Dojindo Laboratories) determined cell apoptosis according to the manufacturer’s procedure. The cells were trypsinized without ethylene diamine tetra acetic acid and suspended in a binding buffer after transfection for 24 and 48 h. The cells were stained with Annexin V-APC for 15 min and subjected to flow-cytometric (FCM) analysis.

### Cell-cycle analysis

The cells were collected at pre-determined intervals, washed twice in phosphate-buffered saline, and fixed in 75% ethanol at 4 °C overnight. Ethanol was removed, and the cells were stained with propidium iodide in RNase staining buffer (BD Pharmingen, San Diego, CA, USA) for 15 min. The BD Biosciences Accuri C6 flow cytometer detected the cell cycle and analyzed it by the Modfit software (LT 5.0, Verity Software House Inc, Topsham, ME, USA).

### Tumor xenografts in vivo

Twenty female BALB/C nude mice (4 weeks, 15–20 g) were purchased from Lingchang Biotech (Shanghai, China). The mice were randomly assigned into shEV (*N* = 10) and shCIRC groups (*N* = 10). Luciferase-labeled shEV or shCIRC RKO cells (4 × 10^6^) were injected subcutaneously, respectively. After 14 days, the tumor volume was measured every 2 days and computed as *V* = (π/6 × length × width^2^). Bioluminescence imaging was performed before sacrificing the mice. Approximately 10 µL/g of 0.7% sodium pentobarbital was injected for anesthetizing. After a few minutes, when animals were anesthetized, they were placed in the living imaging instrument for imaging after injection of D-Luciferin (10 µL/g) for 15 min. The fluorescence was observed and quantitatively analyzed by a small animal living imaging system (Lumina LT, Perkin Elmer, Shanghai, China). All mice were sacrificed by cervical dislocation after anesthesia (when tumor size exceeded 2 cm in length diameter), and the tumors were separated and weighed. Sodium pentobarbital anesthesia was used for all surgical operations, and every attempt was made to reduce animal suffering.

### Statistical analysis

SPSS 22.0 (SPSS Inc., Chicago, IL, USA) was used for statistical analysis. The student’s *t* test (for two groups) or analysis of variance (for multiple groups) evaluated continuous data. Continuous variables were presented as the mean ± standard deviations. Frequency information was provided for categorical variables, and Pearson’s chi-square test was used to assess the information. *P* < 0.05 was used as the statistical significance criterion for all statistical tests.

## Results

### Identification of hsa_circ_0064559 as a potential CRC regulatory factor

**Fig. 1:**
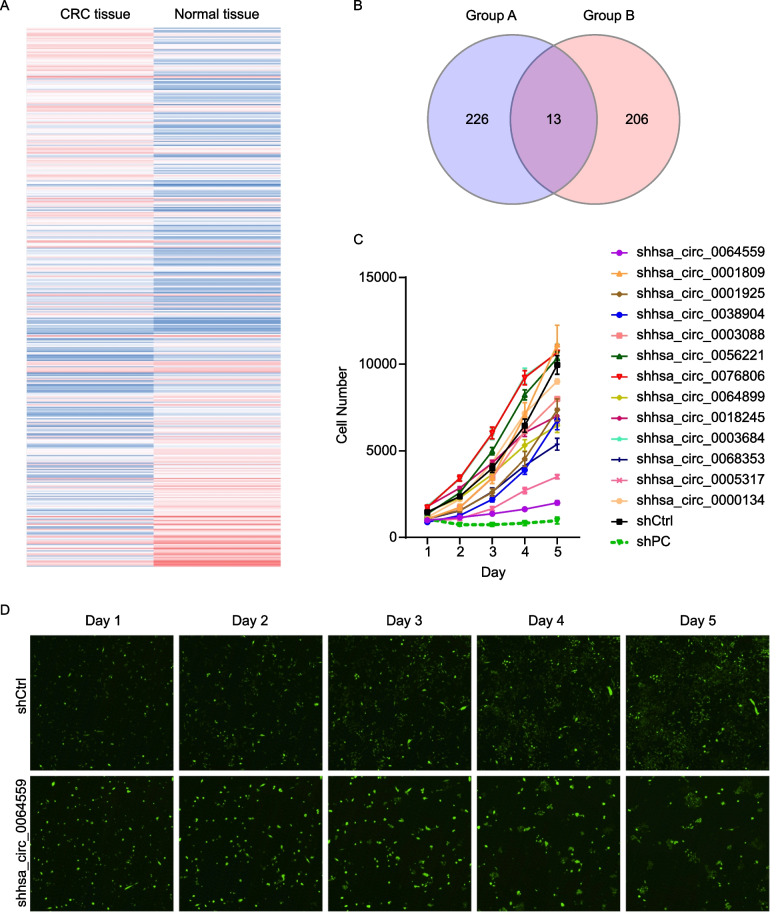
Essential role of hsa_circ_0064559 for the proliferation of colorectal cancer (CRC) cells by Affymetrix Clariom D array. **A** The cluster heat map of the substantially differentially expressed circRNAs (FC > 1.3, *P* < 0.05) between CRC and normal tissues, on a scale of blue (low) to red (high). The columns represent different tissue subsets. **B** Venn diagram showing overlap of upregulated circRNAs in groups A and B. **C** Thirteen genes were selected for validation by high-content screening. shCtrl: non-targeting shRNA, shPC: specific-targeting shRNA (*P < 0.05). **D** Fluorescence staining of CRC cells after knockdown of hsa_circ_0064559 from D1 to D5

Using Affymetrix Clariom D array analysis, we compared the circRNA expression profiles of six pairs of matched CRC and normal colorectal tissue samples in groups A and B to find genes crucial for the development of CRC tumors. According to the data, group A showed two hundred thirty-nine upregulated and one hundred eighty-five downregulated circRNAs, while group B had two hundred nineteen upregulated and one hundred ninety downregulated circRNAs (Fig. 1A). There were thirteen differentially circRNA genes upregulated expression in groups A and B (Fig. 1B). The possible impact on the growth of CRC cells in vitro was investigated by silencing all thirteen candidate genes in RKO cells. Compared with the negative control group, hsa_circ_0064559 knockdown showed the most significant inhibition in cell proliferation (Fig. 1C). Therefore, we concentrated on hsa_circ_0064559 in the following research. As shown in Fig. 1D, the cell proliferation was inhibited in shhsa_circ_0064559 group, especially on D5 after knockdown.

### Knockdown of hsa_circ_0064559 inhibited CRC cell growth in vitro

**Fig. 2:**
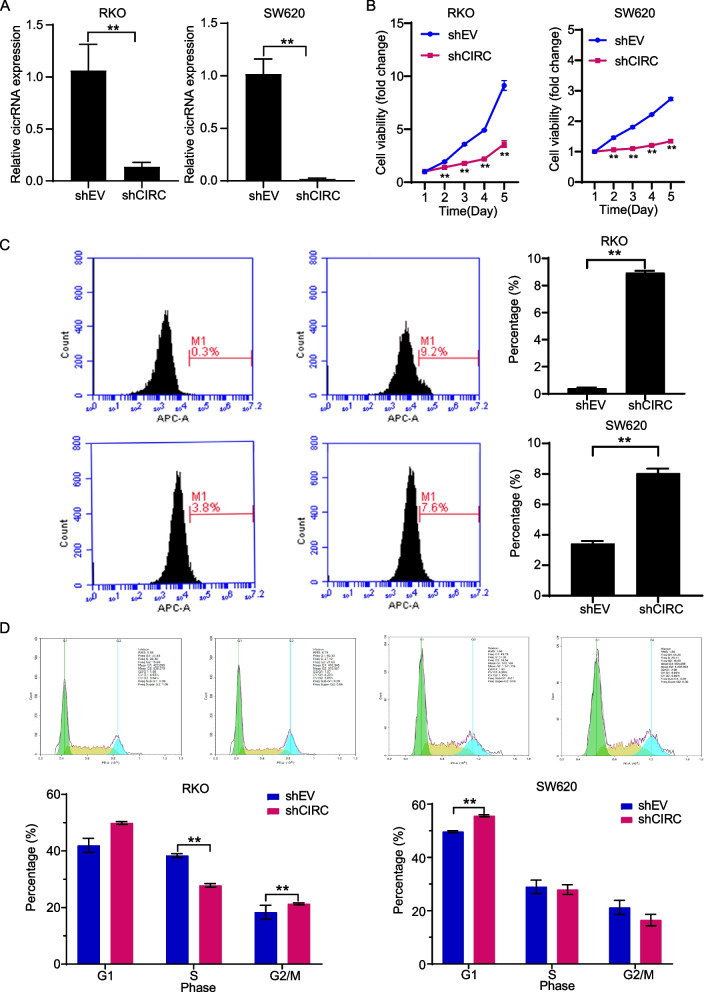
Effects of hsa_circ_0064559 interference on colorectal cancer (CRC) cells in vitro. **A** The expression of hsa_circ_0064559 in RKO and SW620 cell lines was confirmed by real-time quantitative reverse transcription polymerase chain reaction after hsa_circ_0064559 interference (**P < 0.01). **B** The viability of hsa_circ_0064559 knockdown RKO and SW620 cells was detected by 3-(4,5-dimethylthiazol-2-yl)-2,5-diphenyltetrazolium bromide assay (**P < 0.01). **C** The proportion of apoptotic cells in RKO and SW620 cells was stained by Annexin V staining and analyzed by flow-cytometric (FCM) (**P < 0.01). **D** Cell cycle of hsa_circ_0064559 knockdown RKO and SW620 cells was measured by FCM and analyzed by Midfit software (**P < 0.01)

To investigate whether hsa_circ_0064559 influenced CRC tumorigenesis, we performed RNA interference in RKO and SW620 cell lines. Compared to CRC cells transfected with shEV, we discovered that the expression levels of hsa_circ_0064559 were much lower in shCIRC cells (**P < 0.01, Fig. 2A). Subsequently, the results of the MTT assay showed that the knockdown of hsa_circ_0064559 suppressed the growth of CRC cells over five days (**P < 0.01, Fig. 2B). Similarly, after hsa_circ_0064559 knockdown, the percentage of apoptotic cells (APC labeled Annexin V^+^ cells) was increased (**P < 0.01, Fig. 2C). Furthermore, the population of shCIRC cells in G1 phase was increased as analyzed by FCM (**P < 0.01, Fig. 2D). The above findings suggested the regulation role of hsa_circ_0064559 in CRC cell proliferation providing a potential target of CRC.

### Knockdown of hsa_circ_0064559 inhibited the development of CRC tumors in vivo

**Fig. 3:**
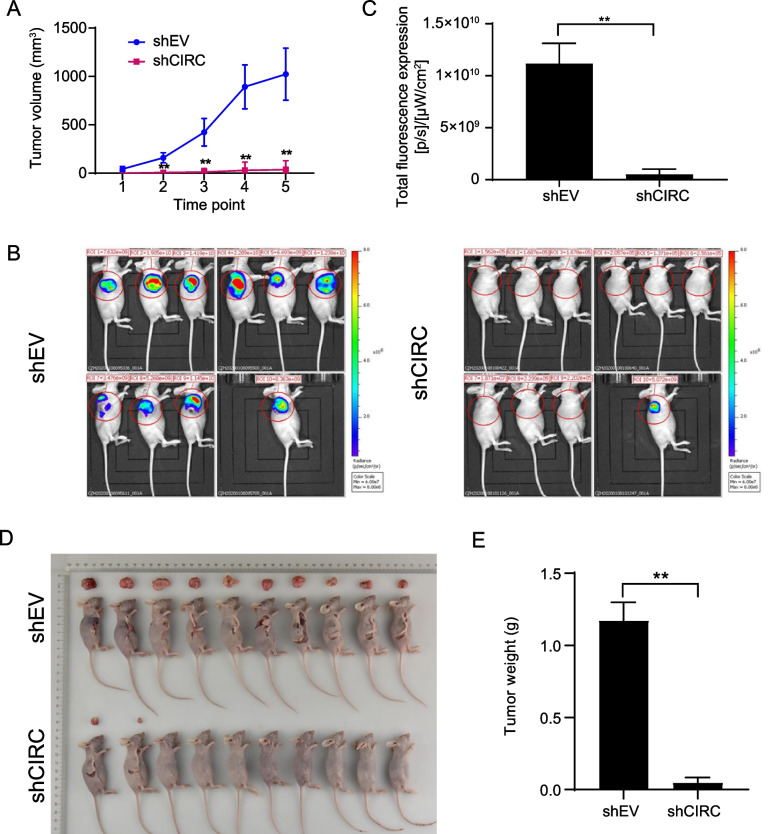
Inhibitory effects of hsa_circ_0064559 interference on developing colorectal cancer (CRC) tumors in vivo. **A** Tumor volume in the shCIRC and shEV groups (**P < 0.01). **B** The bioluminescence imaging in the shEV and shCIRC groups before sacrifice. **C** The overall fluorescence expression in shCIRC and shEV groups (**P < 0.01). **D** The subcutaneous xenograft images of shEV and shCIRC groups. **E** The tumor weight of the two groups (**P < 0.01) (*N* = 10)

We established a xenograft model using shCIRC and shEV transfected RKO cells to confirm the impact of has_ circ_0064559 in vivo. The ShEV group’s tumor volume increased over time. However, the shCIRC group’s tumors hardly developed (**P < 0.01, Fig. 3A). As shown by bioluminescence imaging (Fig. 3B) and total fluorescence expression (Fig. 3C), the tumor volume increased in shEV mice with time, while the tumors hardly formed in shCIRC mice (**P < 0.01). After sacrifice, blatant tumors developed in the shEV group, but only a few were seen in the shCIRC group (**P < 0.01, Fig. 3D and E). The mice with hsa_circ_0064559 knockdown showed significantly decreased tumor volume and weight. These findings suggested that the knockdown of hsa_circ_0064559 could suppress the development of CRC tumors in vivo.

### Expression of involved genes in apoptotic and proliferation pathways

**Fig. 4:**
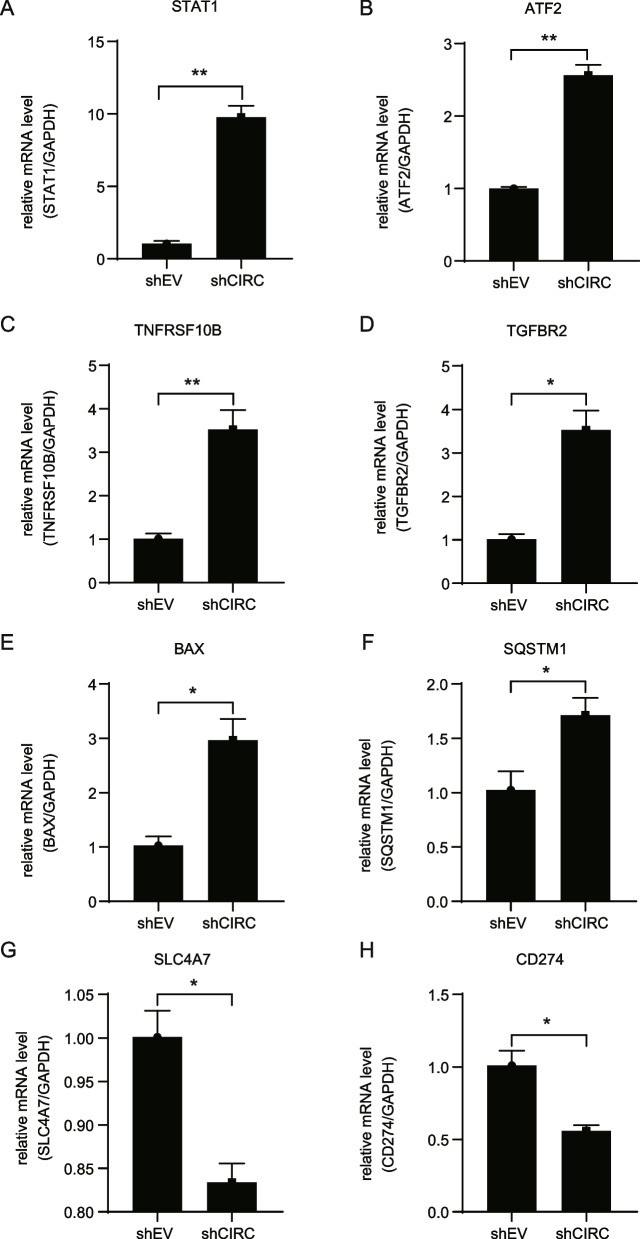
Differential genes’ expression in mRNA levels was verified using real-time quantitative reverse transcription polymerase chain reaction after hsa_circ_0064559 knockdown. **A**–**F** The expression of upregulated genes STAT1, ATF2, TNFRSF10B, TGFBR2, BAX, and SQSTM1 in shCIRC and shEV groups (**P < 0.01, *P < 0.05). **G**,** H** The expression of downregulated genes SLC4A7 and CD274 in shCIRC and shEV groups (*P < 0.05)

To explore the mechanisms of hsa_circ_0064559-induced apoptosis, we performed a human GeneChip Primeview array in two groups of shEV and shCIRC RKO cells. The results showed eight hundred forty-two upregulated genes and one thousand nine downregulated genes after has_circ_0064559 knockdown, among which five hundred and sixty-six genes were related to apoptosis and proliferation of colorectal cancer cells. The results of qRT-PCR showed that eight genes were consistent with the GeneChip results, including six upregulated apoptosis-promoting genes (STAT1, ATF2, TNFRSF10B, TGFBR2, BAX, and SQSTM1) (**P < 0.01, *P < 0.05, Fig. 4A–F) and two downregulated apoptosis-inhibiting gene (SLC4A7, CD274) (*P < 0.05, Fig. 4G and H). The differences in their expression coincided with increased apoptosis and inhibited proliferation by hsa_circ_0064559 knockdown in RKO cells.

## Discussion

Our study investigated the role of circRNA, hsa_circ_006459, in CRC. Our findings demonstrated that hsa_circ_0064559 interference resulted in a decreased rate of proliferation and increased apoptosis in CRC cells. Hsa_circ_0064559 interference suppressed tumor growth in xenograft nude mice. The mechanism of hsa_circ_006459-induced apoptosis demonstrated that its knockdown upregulated the expression of proapoptotic genes and downregulated the expression of inhibitory apoptotic genes. Therefore, hsa_circ_0064559 interference might affect the development of CRC.

CircRNAs form single-stranded closed-loop structures by covalent bonding without 5′ or 3′ free ends [19]. Initially, circRNAs were of little attention and were thought to be by-products of improper alternative splicing. Due to fast advancements in next-generation sequencing methods, several circRNAs have been discovered as functional molecules in disease development rather than splicing by-products [20]. CircRNAs are abundant and conserved RNA molecules with a cell- and tissue-specific spatial expression pattern [21]. CircRNAs play a role in various illnesses, including neurological system disorders, cardiovascular disease, and cancer [22–25]. Several improperly expressed circRNAs have been linked to tumor formation, invasion, metastasis, and patient prognosis [26–28]. Unlike miRNAs and long noncoding RNAs, circRNAs are highly conserved sequences and highly stable in mammalian cells [29]. These characteristics might make circRNAs attractive biomarkers and therapeutic targets for disease detection [30].

CRC development, proliferation, apoptosis, migration, invasion, and metastasis are influenced by circRNAs. Differentially expressed circRNAs are found in drug-resistant CRC cells [31]. CircRNA levels might be employed as indicators for diagnosis, prognosis, and therapeutic targets [32, 33]. In the present study, by comparing the circRNA expression patterns of six matched CRC and normal tissues using Affymetrix Clariom D array analysis, we discovered two hundred thirty-nine upregulated and one hundred eighty-five downregulated circRNAs in group A and two hundred and nineteen upregulated and one hundred ninety downregulated circRNAs in group B. Many circRNAs were differently expressed in CRC versus normal tissues. Zhang et al. used a circular RNA array and detected seventy-six upregulated and one hundred twenty-five downregulated circRNAs in six CRC tissues compared with normal tissues [34]. By comparing four CRC tissue samples with matched normal tissues using RNA-sequencing, Li et al. discovered three hundred ninety-four upregulated and fifty-four downregulated circRNAs [35]. Further screening showed that thirteen genes were co-differentially upregulated in the CRC group compared with normal tissues. HCS screening demonstrated that silencing hsa_circ_0064559 exhibited the most inhibition of proliferation in RKO cells.

We performed RNA interference of thirteen upregulated genes in RKO and SW620 cell lines in vitro. MTT assay revealed that silencing hsa_circ_0064559 decreased the proliferation of CRC cells. When hsa_circ_0064559 was knocked down, the cell apoptosis rate was higher than in the shEV group. Although statistically significant, the proportion of apoptotic cells after silencing was small. However, there is little impact on the finding that hsa_circ_0064559 plays a pivotal role in colorectal cancer growth. In shCIRC transfected cells, FCM revealed a much higher G1 cell population. A small amount of sub-G1 (referring to some fragmented peaks before the cyclical distribution) was observed, resulting from broken DNA appearing in late apoptosis when cells were shattered. It has been demonstrated that several upregulated circRNAs in CRC enhanced cell growth and proliferation. As a result, suppressing their expression limits these activities while encouraging cell death. Zeng et al. discovered that circHIPK3 was upregulated in CRC tissues and HT-29, SW480, and SW620 cell lines. In vitro, CRC cell proliferation, motility, invasion, and induction of apoptosis were hampered by circHIPK3 knockdown. CircHIPK3 acted as a miR-7 sponge, promoting CRC by regulating miR-7 targets, FAK, YY1, IGF1R, and EGFR expression [36]. Yang et al. found that hsa_circ_0004277 expression was upregulated in CRC tissue samples and cell lines, and knocking down hsa_circ_0004277 expedited cell death and restricted CRC cell growth. As a miR-512-5p sponge, hsa_circ_0004277 stimulated the growth of CRC cells by upregulating the expression of human prothymosin alpha (PTMA) [37].

The same results were demonstrated in vivo. The shEV group showed visible tumors in xenograft mice models, but the shCIRC group developed tumors sparingly. Our data demonstrated that knocking down hsa_circ_0064559 might reduce tumor development. Yang et al. demonstrated that hsa_circ_0004277 reduced tumor volume and weight, suppressing CRC cell proliferation in vivo [37]. Zhou et al. found that hsa_circ_0001666 may inhibit CRC cells’ in vivo growth and metastasis [38]. Similarly, suppressing circ-NSD2 inhibited cell migration and invasion of HCT116 and RKO and lung metastasis in animal models [39]. Therefore, circ RNAs may offer therapeutic potential for CRC.

To explore the mechanism by which hsa_circ_0064559 regulates apoptosis and proliferation of CRC cells, we performed Affymetrix GeneChip primeview human gene expression array to analyze the differential expression genes after knockdown of hsa_circ_0064559 expression in RKO cells. According to the differential expression ratio, five hundred sixty-six different expression genes related to apoptosis and proliferation of CRC cells were divided into high, middle, and low sections. Each section randomly selected five genes, and qRT-PCR verified their expressions. The results of qRT-PCR showed that eight genes were consistent with the GeneChip results. The expressions of STAT1, ATF2, TNFRSF10B, TGFBR2, BAX, and SQSTM1 were upregulated. STAT1 is a tumor suppressor whose activation could lead to the overproduction of oxygen species and tumor cell apoptosis [40]. ATF2 has several functions in different cancers. Several studies have demonstrated low ATF2 levels correlated with poor prognosis and tumor aggressiveness in CRC patients [41]. TNFRSF10B (TRAILR2) is a death receptor on the cell surface activated by binding to tumor necrosis factor-related apoptosis-inducing ligand (TRAIL), p53-induced DNA damage, or C/EBP homologous protein-induced endoplasmic reticulum stress and induces the apoptosis of tumor cells [42]. TGF-beta receptor II (TGFBR2) was a tumor suppressor in CRC [43]. BAX is an apoptosis-promoting gene in the BCL-2 gene family, and its overexpression can antagonize the protective effects of BCL-2 and induce the apoptosis of tumor cells [44]. It has been shown that the ubiquitin-binding protein p62/SQSTM1 promotes death receptor-induced cell death in CRC [45]. Meanwhile, the expression of SLC4A7 and CD274 were downregulated. SLC4A7 (also called NBCn1) is widely expressed in several tissues and serves as a Na^+^/HCO_3_^−^ cotransporter. The research has revealed that SLC4A7 promotes the occurrence and development of breast cancer, and its high expression is related to the poor prognosis of patients [46]. CD274 (PD-L1), expressed on the surface of tumor cells, is a ligand of PD-1, which can transmit inhibitory signals and mediate the immune escape effect of tumor cells. The blocking of the PD-1/PD-L1 axis showed great potential in tumor therapy [47]. All eight differential expressed genes after hsa_circ_0064559 knockdown induced the apoptosis of tumor cells and inhibited tumor development.

CircRNAs have unique properties that make them ideal candidates for biomarkers, including their abundance, stability, cross-species conservation, and disease-specific and dynamic expressions. CircRNA levels might be utilized as indicators for diagnosis and prognosis. Hsa_circ_0001178 [32], circITGA7 [48], hsa_circ_0000567 [49], hsa_circ_0001649 [50], hsa_circ_0000826 [32], and hsa_circ_0000711 [51] were circRNAs that had an area under the curve (AUC) greater than 0.8 for diagnosing CRC (with circ 0001178 having an AUC of 0.945), implying their excellent diagnostic accuracy as CRC biomarkers. The limitation of our study is that we investigated the effects of hsa_circ_0064559 on the proliferation and apoptosis of CRC cells in vitro and in vivo without addressing the specific mechanisms and exploration of hsa_circ_0064559 as a clinical tumor marker. Further evaluation of the potential therapeutic effects of hsa_circ_0064559 is required, particularly for treating existing tumors. Further studies on the mechanism may provide more comprehensive conclusions.

## Conclusions

The present study suggested that hsa_circ_0064559 is upregulated in CRC tumor tissues that promote apoptosis by regulating the expression of apoptosis-related genes, thus playing a critical role in tumorigenesis. These findings indicated the possibility of developing hsa_circ_0064559 as a clinical biomarker and potential drug target. Our results provided novel insights into the therapeutic strategy suppressing CRC progression.

## Data Availability

The datasets used and/or analyzed in the present study are available from the corresponding author upon reasonable request.

## Abbreviations

CRC: Colorectal cancer
circRNAs: Circular RNAs
DMEM: Dulbecco’s modified Eagle medium
shCIRC: Short hairpin RNA targets for hsa_circ_0064559
shEV: Empty vectors
qRT-PCR: Quantitative real-time polymerase chain reaction
HCS: High-content screening
FCM: Flow-cytometric
PTMA: Prothymosin alpha
AUC: Area under the curve
TNM: Tumor-node-metastasis
